# The diverse *N*-glycosylation profiles of CD4^+^CD25^-^ and CD4^+^CD25^+^ T cells in Hashimoto’s thyroiditis

**DOI:** 10.3389/fimmu.2025.1633344

**Published:** 2025-09-15

**Authors:** Sara Trzos, Marta Szewczyk, Paweł Link-Lenczowski, Grzegorz Sokołowski, Małgorzata Trofimiuk-Müldner, Katarzyna Bocian, Ewa Pocheć

**Affiliations:** ^1^ Department of Glycoconjugate Biochemistry, Institute of Zoology and Biomedical Research, Faculty of Biology, Jagiellonian University, Krakow, Poland; ^2^ Doctoral School of Exact and Natural Sciences, Faculty of Biology, Jagiellonian University, Krakow, Poland; ^3^ Department of Medical Physiology, Faculty of Health Sciences, Jagiellonian University Medical College, Krakow, Poland; ^4^ Center for the Development of Therapies for Civilization and Age-Related Diseases, Jagiellonian University Medical College, Krakow, Poland; ^5^ Department of Endocrinology, Faculty of Medicine, Jagiellonian University Medical College, Krakow, Poland; ^6^ Department of Immunology, Institute of Experimental Zoology, Faculty of Biology, University of Warsaw, Warsaw, Poland

**Keywords:** CD4^+^ T cells, *N*-glycosylation, *N*-glycans, glycosyltransferases, Hashimoto’s thyroiditis, autoimmunity, L-thyroxine

## Abstract

Hashimoto’s thyroiditis (HT) is one of the most common organ-specific autoimmune diseases, characterized by chronic thyroid gland inflammation. Helper T (Th) CD4^+^ cells, whose surface receptors are highly glycosylated, are involved in the pathomechanism of HT. Our study aimed to characterize *N*-glycosylation profiles in two pools of CD4^+^ T cells, defined by the expression of CD25^+^ late activation marker (CD4^+^CD25^+^) and CD25-negative cells (CD4^+^CD25^-^) in HT. Two study groups were recruited: HT1 with elevated thyroid autoantibodies and TSH level within the normal range without hypothyroidism, and HT2, hypothyroid HT patients, adequately metabolically controlled while on L-thyroxine replacement therapy, and healthy subjects to the control group (CTR). *N*-glycans from CD4^+^ cell proteins, released using *N*-glycosidase F, were analyzed by MALDI-Tof mass spectrometry. RT-qPCR was used to determine the expression of selected glycogenes. We found significant differences in the glycome of CD4^+^CD25^-^ and CD4^+^CD25^+^ cells. In homeostasis (CTR), a predominance of complex-type glycans was observed in CD4^+^CD25^-^ cells, whereas the oligomannose-type structures prevail in CD4^+^CD25^+^ lymphocytes. In autoimmunity and progressive thyroid dysfunction, the rearrangement of *N*-glycans in Th cells was observed, in opposite directions in the CD4^+^ pools. Complex-type structures are replaced by oligomannose forms in CD4^+^CD25^-^ in the HT1 group, while in HT2, a restoration of glycosylation profile to the level of CTR was detected. CD4^+^CD25^+^ cells accelerated complex-type synthesis in HT1, which was normalized in HT2 patients. Changes in the profile of *N*-linked glycans are partially reflected in the expression of mannosidases and glycosyltransferases. Our study demonstrates for the first time the diverse *N*-glycosylation profiles in CD4^+^CD25^-^ and CD4^+^CD25^+^ cells, and the rearrangement of *N*-glycan structures specific for each pool of Th cells in HT. Further studies are needed to determine the functional aspect of the identified *N*-glycosylation changes during thyroid autoimmunity.

## 1 Introduction

Hashimoto’s thyroiditis (HT), also known as lymphocytic thyroiditis, is an organ-specific autoimmune disease. It is characterized by a breakdown of self-tolerance to thyroid antigens, resulting in circulating autoantibodies to thyroperoxidase (TPO) and thyroglobulin (Tg) and lymphocytic infiltration of the gland (1). As a consequence, there is the destruction of thyrocytes and thyroid follicles, resulting in a decrease of thyroid hormone production (hypothyroidism) in some patients (2).

The pathogenesis of HT is essentially related to a cellular-type immune response, but, as in other autoimmune diseases, the cellular and humoral responses are closely related (3). An important step in thyroid autoimmunity is the accumulation of antigen-presenting cells (APCs), mainly macrophages, which express the major histocompatibility complex (MHC) class II. Additionally, in HT patients, thyrocytes abnormally express MHC II, which presents thyroid autoantigens and, as a result, facilitates T cell differentiation and activation (4, 5). APCs activate naïve T cells and lead to their differentiation into three subsets of T helper (Th) cells: Th1, Th2, and Th17. In HT, Th1 cells secrete pro-inflammatory cytokines: interferon-gamma (IFNγ) and tumor necrosis factor-alpha (TNFα) that activate cytotoxic T cells (Tc) and macrophages, leading to inflammation and damage to the gland. Interleukins (IL)-4, -5, and -10, produced by Th2 lymphocytes, stimulate B cells to differentiate into plasmocytes, which secrete antibodies directed against thyroid autoantigens. As a result, the autoantibodies induce the destruction of thyrocytes by antibody-dependent cellular cytotoxicity (ADCC) or complement-dependent cytotoxicity (CDC). Additionally, Th17 cells secrete IL-17, which stimulates epithelial cells and fibroblasts to produce pro-inflammatory cytokines, triggering apoptosis of thyroid follicle cells (5–7).

A rising number of studies indicate that *N*-glycans on T cell receptors, including CD4 and CD25, are one of the key regulators of their biology and functions. *N*-glycans are formed and attached to proteins by a post-translational enzymatic process called *N*-glycosylation. Glycans affect thymocyte selection, proliferation, differentiation, and activation of T cells. These effects underscore the importance of glycans as determinants of T cell self-tolerance or hyperreactivity, which may ultimately be linked to loss of immune tolerance in autoimmune diseases (8).

The role of T cell *N*-glycans has been studied in patients with ulcerative colitis (UC). Differences between the active and inactive forms of UC in the content of the β1,6-GlcNAc (*N*-acetylglucosamine)-branched complex type *N*-oligosaccharides on Th cells depended on the severity of the disease. The lowest level of β1,6-GlcNAc was observed in patients with severe disease symptoms. The results correlated with the expression of the 6-β-*N*-acetylglucosaminyltransferase gene *MGAT5* responsible for β1,6-GlcNAc attachment, where those with active UC had lower levels of *MGAT5* transcript compared to healthy subjects (9, 10).

Based on the above rationale, our study aimed to determine changes in CD4^+^ T cell *N*-glycosylation in HT and verify if these alterations correspond to the severity of the thyroid dysfunction. We used matrix-assisted laser desorption/ionization mass spectrometry with a time-of-flight analyzer (MALDI-Tof MS) for quantitative and qualitative analysis of Th cell *N*-glycans from subjects showing high autoantibody titers (HT1) with non-affected TSH level, and HT patients after stabilization of TSH level due to L-thyroxine therapy (HT2), compared to healthy donors (CTR). We also analyzed the differences in the gene expression of selected α-mannosidases and glycosyltransferases by RT-qPCR.

## 2 Materials and methods

### 2.1 Characteristics of the study and control groups

Recruitment of donors with HT was carried out through verification of donor status by an endocrinologist at the University Hospital in Krakow. The study group included adult patients with increased concentrations of TPOAb and/or TgAb, and characteristic features of HT on thyroid ultrasound and medical history. Exclusion criteria were other chronic diseases, alcohol abuse, and pregnancy, as all of the above factors affect the glycosylation process (11, 12).

**Figure 1:**
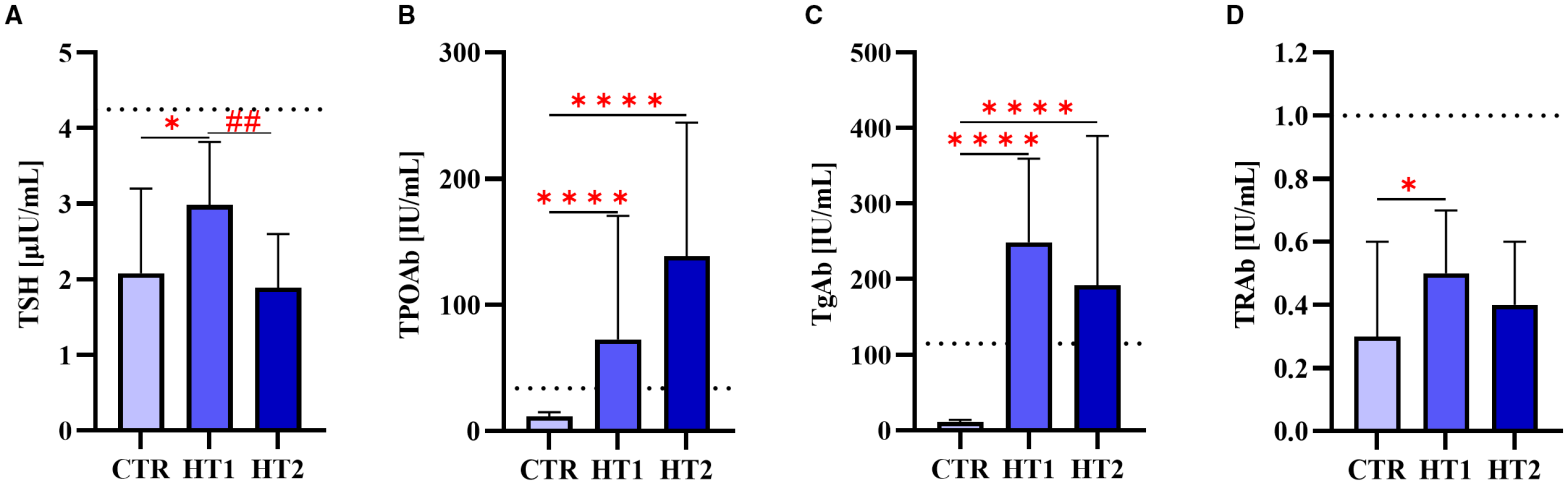
The levels of **(A)** thyroid-stimulating hormone (TSH) and autoantibodies: **(B)** against thyroperoxidase (TPOAb), **(C)** thyroglobulin (TgAb), **(D)** TSH receptor (TRAb) in donors showing high levels of autoantibodies (HT1), patients with Hashimoto’s thyroiditis during L-thyroxine therapy (HT2), and healthy donors (CTR). Results were expressed as median ± IC. The dotted line indicates the upper normal limit of TSH and autoantibodies. The statistical significance of the data was assessed using the Kruskal-Wallis test with Dunn’s correction. Significance levels between the study and control groups are marked with asterisks as follows: * p ≤ 0.05 and **** p ≤ 0.0001. HT2 vs. HT1 comparisons are marked with crosses as follows ## p ≤ 0.01.

Within the study group, two subgroups were distinguished: HT1 (n=45), donors with elevated concentrations of TPOAb and/or TgAb autoantibodies and TSH level within the normal range without symptoms of hypothyroidism, and HT2 (n=60), hypothyroid HT patients, adequately metabolically controlled while on L-thyroxine replacement therapy. Healthy subjects were recruited to the control group (CTR, n=53) based on TSH level and antithyroid antibodies (TPOAb, TgAb, and TRAb) within the normal range, as well as normal thyroid ultrasound. The characteristics of the study and control groups are shown in **Table 1** and **Figure 1**.

**Table 1:** Characteristics of healthy subjects (CTR, control group), donors with high autoantibody titers and TSH levels in the normal range (HT1), and patients with Hashimoto's thyroiditis during L-thyroxine therapy (HT2) (study groups).

Group	Sex (F)	Age	TSH (0.27-4.20 mIU/ml)	TPOAb (<34.0 IU/ml)	TgAb (<115.0 IU/ml)	TRAb (0.0-1.0 IU/ml)
CTR	53	18-46 33.06 ± 8.56	2.44 ± 1.13	13.38 ± 6.61	16.00 ± 14.78	0.40 ± 0.24
HT1	45	18-45 32.34 ± 8.65	3.23 ± 2.02	126.51 ± 163.21	268.14 ± 219.23	0.58 ± 0.36
HT2	60	20-45 34.42 ± 7.28	2.41 ± 2.18	145.18 ± 144.12	230.73 ± 228.25	1.35 ± 4.51

Mean ± SD is given for age, antibody levels, and TSH. TPOAb, anti-thyroperoxidase antibodies; F, female; TgAb, anti-thyroglobulin antibodies; TRAb, antibodies directed against the receptor for thyroid-stimulating hormone; TSH, thyroid-stimulating hormone.

### 2.2 Isolation of CD4^+^CD25^-^ and CD4^+^CD25^+^ T cells

#### 2.2.1 Isolation of PBMCs

**Figure 2:**
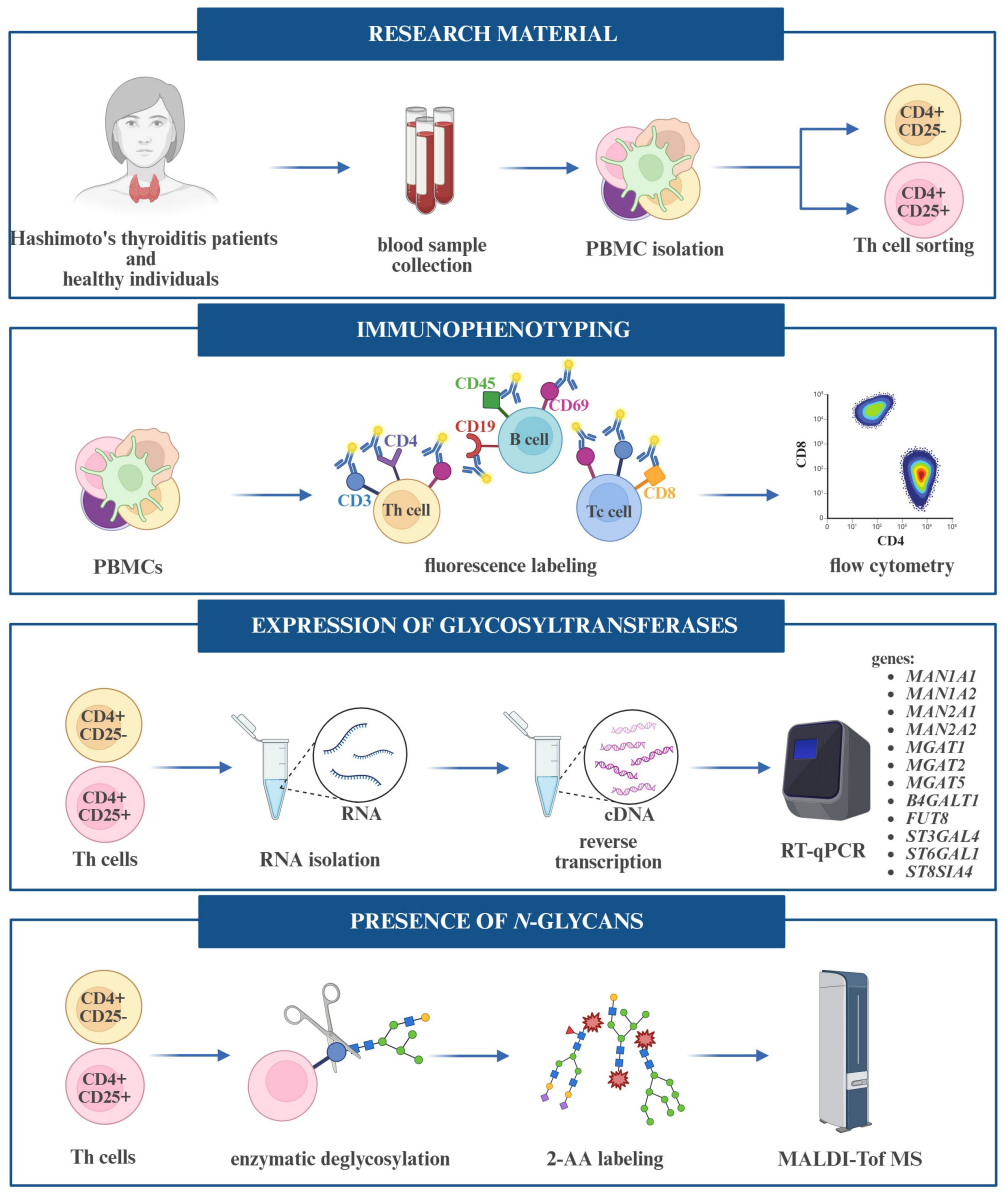
Workflow of experiments performed on peripheral blood mononuclear cells (PBMCs) and T helper (Th) cells.

Blood from HT patients and healthy subjects was collected into a tube with EDTA as an anticoagulant. Peripheral blood mononuclear cells (PBMCs) were isolated from 20 mL of whole blood, which was diluted into 35 mL Running Buffer (PBS pH 7.2 with BSA, EDTA, and 0.09% azide) (130-091-221, Miltenyi Biotec, Teterow, Germany). Diluted blood was layered on 15 mL Histopaque-1077 (10771, Sigma-Aldrich, Saint Louis, MO, USA) and centrifuged at 1500 rpm for 25 min at room temperature (RT). The density gradient centrifugation resulted in a layer of PBMCs at the interface between serum and Histopaque-1077. The cell suspension was then topped up with a Running Buffer to 50 mL and washed two times by centrifugation (1900 rpm, 10 min, RT). Cells were resuspended in 2.5 mL Running buffer, and their quantities were counted using a Bürker chamber after a 20-fold dilution in PBS. The stages of conducting the experiments are shown in **Figure 2**.

#### 2.2.2 CD4^+^ T cells sorting

PBMCs and CD4^+^ T cells were labeled according to the manufacturer’s leaflet, and the volumes of reagents were calculated based to the number of isolated cells.

After re-centrifugation (1900 rpm, 10 min, RT) to remove the buffer excess, PBMCs were suspended in an appropriate volume of Running Buffer. CD4^+^ T Cell Biotin-Antibody Cocktail (CD4^+^ T cell Isolation Kit human, 130-096-533, Miltenyi Biotec) was added and incubated for 5 min at 4 °C. Another incubation was carried out after adding Running Buffer and CD4^+^ MicroBead Cocktail (CD4^+^ T target Isolation Kit human, 130-096-533, Miltenyi Biotec) for 10 min at 4 °C. Samples were subjected to sorting in an autoMACS Pro Separator magnetic sorter (Miltenyi Biotec). After sorting, fractions of CD4^+^ and CD4^-^ cells were obtained, and their number was determined using a TC20 Automated Cell Counter (Bio-Rad) after a 5-fold dilution of cells in PBS.

#### 2.2.3 CD4^+^CD25^-^ and CD4^+^CD25^+^ T cells sorting

CD4^+^ T cells were centrifuged at 1900 rpm for 10 min at RT after sorting, resuspended in Running Buffer, and CD25 MicroBeads II (130-092-983, Miltenyi Biotec) were added. Incubation was carried out for 15 min at 4 °C, the Running Buffer was added, and cells were centrifuged again (1900 rpm, 15 min, 4 °C). In the last step, cells suspended in the appropriate volume of buffer were sorted in an autoMACS Pro Separator magnetic sorter (Miltenyi Biotec). The number of isolated CD4^+^CD25^-^ and CD4^+^CD25^+^ cells was determined using a cell counter (Bio-Rad). Selected lymphocyte pellets were resuspended in RLT buffer (79216, Qiagen, Hilden, Germany) for RNA isolation (RT-qPCR) or allowed to homogenize for protein extraction (MALDI-ToF MS).

#### 2.2.4 Assessment of the purity of Th cell subpopulations

CD4^+^CD25^-^ and CD4^+^CD25^+^ T cells were checked for purity by flow cytometry (FACSCalibur, Becton Dickinson, East Rutherford, NJ, USA). The 3x10^5^ isolated cells were prepared for flow cytometry analysis by labeling with anti-CD4 IgG conjugated with peridinin-chlorophyll-protein complex (PerCP) (BD Pharmingen™ PerCP Mouse Anti-Human CD4) and anti-CD25 IgG conjugated with allophycocyanin (APC) (BD™ APC Mouse Anti-Human CD25). The obtained results were processed using BD FACSDiva software.

### 2.3 Immunophenotyping of lymphocytes

Th, Tc, and B lymphocyte subpopulations were identified among isolated PBMCs by flow cytometry. For this purpose, BD anti-CD4 FITC/CD69 PE/CD3 PerCP (Th cells), anti-CD8 APC (Tc cells), and anti-CD19 FITC/CD69 PE/CD45 PerCP (B cells) immunophenotyping kits were used according to the manufacturer’s protocols. The obtained results were analyzed using BD FACSDiva software.

### 2.4 MALDI-ToF MS in negative mode

#### 2.4.1 Preparation of cellular protein extracts

Both CD4^+^ T cell pools were homogenized in 250 μL of radioimmunoprecipitation (RIPA) buffer (89900, Thermo Fisher, Waltham, MA, USA) with protease inhibitors (P8340, Thermo Fisher). The samples were then incubated on ice for 10 min and centrifuged at 18000 rpm at 4 °C for 15 min. The collected lysates were chemically precipitated using chloroform (372978, Sigma-Aldrich) and methanol (MeOH, 100837, Sigma-Aldrich). To 100 μL of lysate, 400 μL of MeOH, 100 μL of chloroform, and 300 μL of deionized water (MQ) were added sequentially, each time mixing the solutions. The samples were then centrifuged at 15000 rpm for 2 min. After the filtrates were discarded, the precipitated protein was collected, and 400 μL of MeOH was added, and centrifuged again under the same conditions (15000 rpm, 2 min). The MeOH was collected, and the pellet was allowed to dry.

#### 2.4.2 Enzymatic deglycosylation of proteins

The dried protein pellets were suspended in 20 μL of 10x concentrated denaturation buffer (5% sodium dodecyl sulfate (SDS), 0.4 M dithiothreitol (DTT) (B1704S, New England Biolabs, Ipswich, MA, USA) and incubated for 10 min at 100 °C. After cooling the samples, 20 μL of the buffer consisting of 10x concentrated Glycobuffer 2 (50 mM sodium phosphate, pH 7.5) (B3704S, New England Biolabs), 10x concentrated NP-40 (B2704S, New England Biolabs), and MQ was added, and finally 1.5 μL of PNGase F (500000 U/mL, P0705L, New England Biolabs). Samples were incubated overnight at 37 °C. Released *N*-glycans were desalted by solid phase extraction (SPE) on Supelclean™ ENVI-Carb™ SPE columns (57109-U, Sigma-Aldrich) according to the method described by Packer et al. (13), and the eluted *N*-glycans were dried by lyophilization (Labconco).

#### 2.4.3 2-AA labeling of *N*-glycans

Appropriate amounts of 2-aminobenzoic acid (2-AA) (PP0530, Sigma-Aldrich) and sodium cyanoborohydride (156159, Sigma-Aldrich) were dissolved in dimethylsulfoxide (DMSO) (D4942, Sigma-Aldrich) and acetic acid in a 3:2 ratio. The mixture (30 μL) was added to concentrated *N*-glycan samples and incubated for 3 hours at 65 °C. According to the manufacturer’s instructions, the excess tracer was removed using Spe-ed Amide-2 columns (4821, Applied Separation, Allentown, PA, USA). The collected fractions were concentrated to dryness (Labconco).

#### 2.4.4 MALDI-Tof MS analysis

Concentrated and labeled *N*-glycans were dissolved in 10 μL of 0.1% trifluoroacetic acid (TFA) in MQ and desalted using a C18ZipTip™ (ZTC 18S096, Millipore, Darmstadt, Germany) according to the manufacturer’s instructions. *N-*oligosaccharides were eluted with a 10 mg/mL solution of 2,5-dihydroxybenzoic acid dissolved in a 50% aqueous acetonitrile solution containing 0.1% TFA in a 1:1 ratio directly onto an MTP 384 ground BC steel target plate (8280784, Bruker Daltonics, Bremen, Germany). The applied samples were then dried at RT.

Analysis was performed on a rapifleX™ mass spectrometer (MALDI-Tof) controlled by FlexControl software (Bruker Daltonics). The instrument was externally calibrated using the Bruker Peptide Calibration Standard II (8206195, Bruker Daltonics). Mass spectra were in negative ion reflectron mode in the *m/z* range of 700 to 5000 for 32000 shots/sample. *N-*glycans peaks were described based on *m/z* values, and graphical structures were created using GlycoWorkbench software (version 2.1, European Carbohydrates DataBase project; http://www.eurocarbdb.org/).

### 2.5 Expression analysis of glycogenes by RT-qPCR

Total cellular RNA was isolated by the columnar method using the RNeasy Plus Mini Kit (74134, Qiagen) for CD4^+^CD25^-^ T cells, and the RNeasy Plus Micro Kit (74034, Qiagen) for CD4^+^ CD25^+^ T cells. The isolated RNA (0.5 – 1 μg) was reverse transcribed into cDNA using the High-Capacity RNA-to-cDNA Kit (4387406, Applied Biosystems, Foster City, CA, USA). The cDNA was subjected to RT-qPCR using TaqMan Gene Expression Master Mix (4444557, Applied Biosystems) to determine the expression of genes encoding glycosyltransferases: α1,6-fucosyltransferase 8 (*FUT8*), 6-β-*N*-acetylglucosaminyltransferase (*MGAT5*), β-galactoside α2,3-sialyltransferase 4 (*ST3GAL4*), β-galactoside α2,6-sialyltransferase 1 (*ST6GAL1*), and α2,8-sialyltransferase 4 (*ST8SIA4*) (Applied Biosystems). The mRNA amplification of ribosomal protein lateral stalk subunit P0 (*RPLP0*) and ribosomal protein L13a (*RPL13a*) (Applied Biosystems) was used as an internal control for reaction efficiency. Probe IDs are given in **Table 2**. cDNA was also subjected to RT-qPCR using PowerUp™ SYBR™ Green Master Mix (A25742, Applied Biosystems) to determine the expression of genes encoding the glycosidases: α1,2-mannosidase IA (*MAN1A1*), α1,2-mannosidase IIA (*MAN1A2*), α1,3/1,6-mannosidase IA (*MAN2A1*) and α1,3/1,6-mannosidase IIA (*MAN2A2*), and the glycosyltransferases: β1,4-galactosyltransferase 1 (*B4GALT1*), α1,3-mannosyl-2-β-*N*-acetylglucosaminyltransferase (*MGAT1*), and α1,6-mannosyl-2-β-*N*-acetylglucosaminyltransferase (*MGAT2*) (Genomed, Warsaw, Poland). RPLP0 mRNA amplification was used as an internal control for reaction efficiency. The sequences of the primers are given in **Table 3**.

**Table 2:** Probe IDs of selected glycosyltransferases and housekeeping genes.

Gene abbreviation	Probe ID
*FUT8*	Hs00189535_m1
*MGAT5*	Hs00159136_m1
*ST3GAL4*	Hs00920870_m1
*ST6GAL1*	Hs00949382_m1
*ST8SIA4*	Hs00379924_m1
*RPL13a*	Hs04194366_m1
*RPLP0*	Hs99999902_m1

**Table 3:** Primer sequences of selected glycosidases and glycosyltransferases, and the housekeeping gene.

Gene abbreviation	Forward	Reverse
*B4GALT1*	CCTCTTGCCTGTCCCCTAAA	GCACCTGTGAAAACCTGAGG
*MAN1A1*	TGTGACAGCCTCCTTATGCA	TCCAGAAATGACACAGGGCA
*MAN1A2*	GGAGGGGAAGCCTGTATTCA	GGTGTGTGTGTTTGATCCCC
*MAN2A1*	GCTGCTTCCTAGAGTCCACA	CAGCATCAGGTAGAGCGAGA
*MAN2A2*	GGCCCTTTCTTCTCAGAGGT	AGATACCCTGGCTGTCGATG
*MGAT1*	CCTGGAGAGCAACTGAGACA	AGAAGAAGAGGAGCAGCAGG
*MGAT2*	CCCGAATACCTCAGACTGCT	AAAGAACACCTGCAGAACCG
*RPLP0*	ATGGCAGCATCTACAACCCT	AGGACTCGTTTGTACCCGTT

### 2.6 Statistical analysis

The *N*-glycan analysis is presented as the abundance of each glycan species, which was normalized to the sum of the peak areas of all glycans detected by MALDI-Tof MS analysis and presented as percentages of total glycan abundance. Results from RT-qPCR are presented as 2^-ΔΔCt^, and reaction yields were calculated using PCR Miner software (http://miner.ewindup.cn/miner/), developed by Zhao and Fernald (14). The non-parametric Kruskal-Wallis test with Dunn’s correction was used for blood test data, MALDI-Tof MS and RT-qPCR results, and two-way ANOVA with Bonferroni correction was applied for a comparison between both CD4^+^ cell pools. P<0.05 was considered significant. Statistical analysis was performed using GraphPad Prism 9 software.

## 3 Results

**Figure 3:**
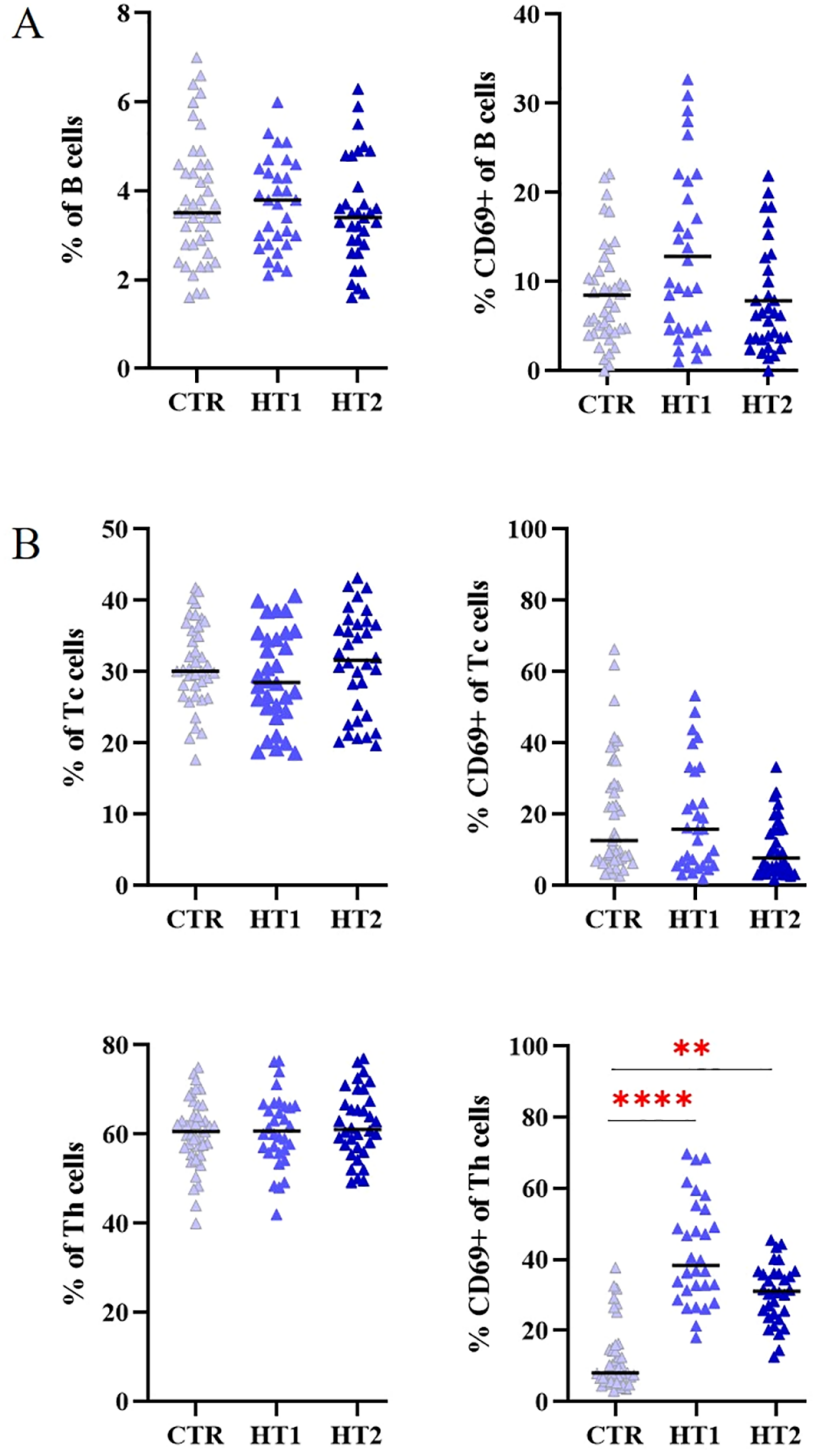
Flow cytometry analysis of **(A)** B, and **(B)** Tc, Th lymphocytes from healthy donors (CTR, control group) and patients with high autoantibody titers and TSH levels in the normal range (HT1) and patients with Hashimoto’s thyroiditis during L-thyroxine therapy (HT2) (study groups). The percentages of the entire pool of lymphocytes and among them those expressing the early activation marker CD69, are shown as mean values ± SD. The statistical significance of the data was assessed using the Kruskal-Wallis test with Dunn’s correction, and significance levels between the study and control groups are marked with asterisks as follows: **p ≤ 0.005, and ****p ≤ 0.0001.

**Figure 4:**
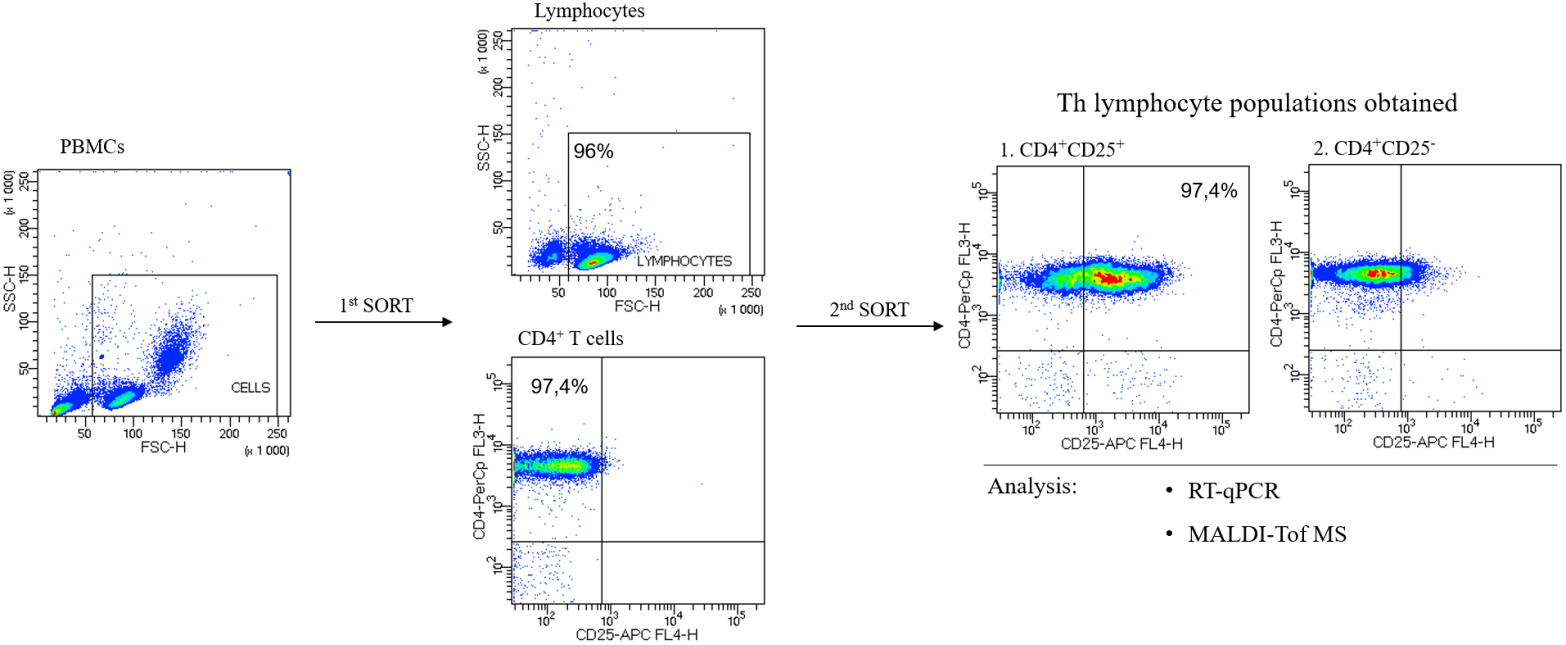
Representative flow cytometric analysis of the purity of T helper (Th) cells sorted from human peripheral blood. In the first step, CD4^+^ cells were sorted from peripheral blood mononuclear cells, PBMCs (1^st^ SORT), and in the 2^nd^ SORT, the populations of CD4^+^CD25^-^ and CD4^+^CD25^+^ cells were obtained from CD4^+^ T cells. Both pools of Th cells were analyzed by RT-qPCR and MALDI-Tof MS.

For quantitative and qualitative comparisons of the main lymphocyte subpopulations between the study (HT1 and HT2) and control (CTR) groups, immunophenotyping was performed by flow cytometry (**Figure 3**). Isolated PBMCs were analyzed using fluorochrome-conjugated antibodies recognizing specific lymphocyte surface receptors: CD3^+^CD4^+^ for Th cells, CD3^+^CD8^+^ present on Tc cells, and CD19^+^CD45^+^ specific for B cells. In addition, the pool of activated cells (CD69^+^) was assessed in each of these subtypes (**Figure 3**). An increase in the percentage of Th cell subpopulations with the activation marker CD69 was observed in both HT1 and HT2 patients compared to healthy subjects. A decreasing trend was noted in the population of CD3^+^CD4^+^CD69^+^ cells in HT2 vs. HT1, but the result was not statistically significant (**Figure 3B**). The efficiency of the isolation into CD4^+^ (1^st^ SORT), and CD4^+^CD25^-^ and CD4^+^CD25^+^ (2^nd^ SORT) subpopulations was confirmed by flow cytometry (**Figure 4**). Both CD4^+^ cell pools were subjected to MALDI-Tof MS and RT-qPCR analysis.

**Figure 5:**
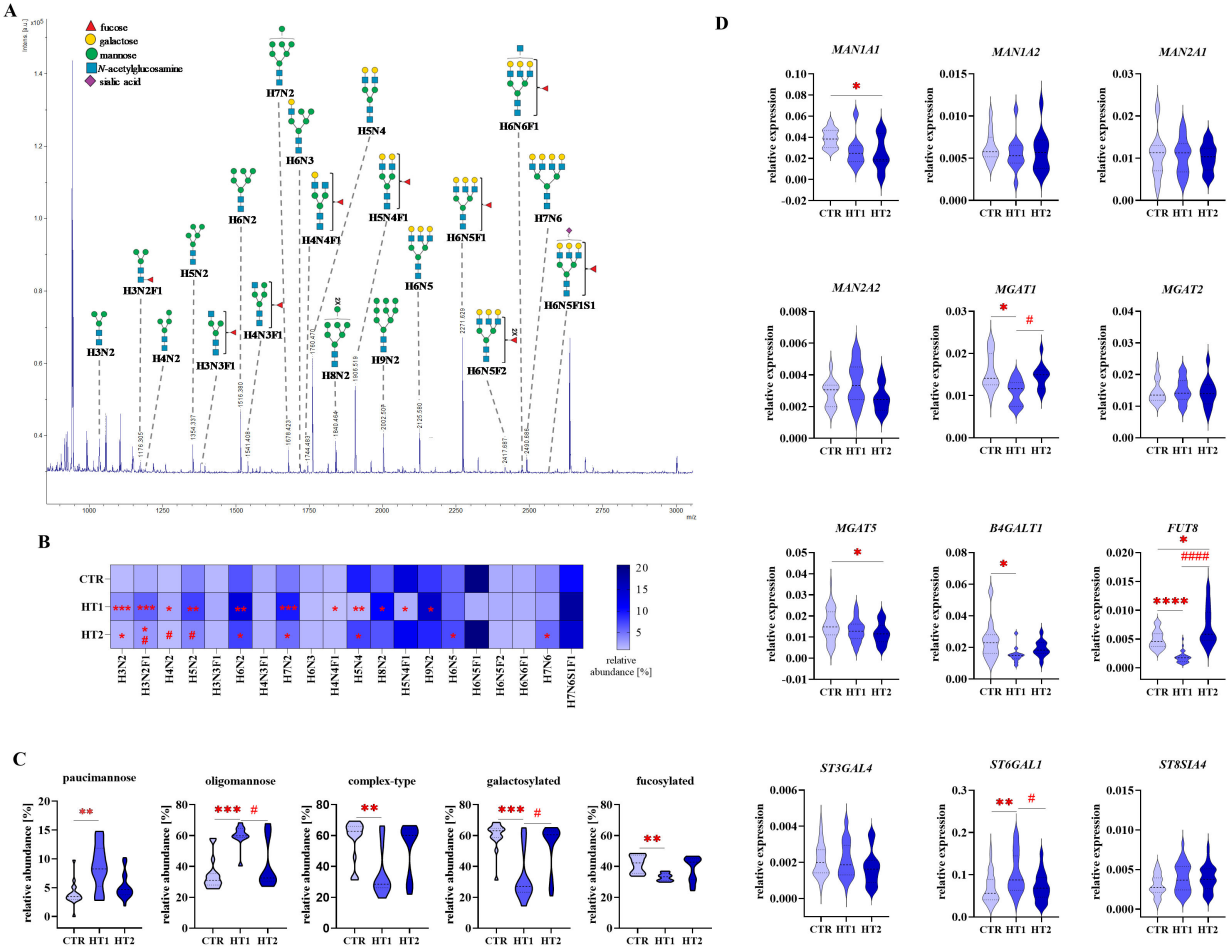
Analysis of *N*-glycan structures by MALDI-Tof MS and glycogene expression analysis by RT-qPCR in CD4^+^CD25^-^ Th cells from donors with high titers of autoantibodies without symptoms of hypothyroidism (HT1) and patients with Hashimoto’s thyroiditis during L-thyroxine therapy (HT2) and from healthy donors (CTR). *N*-glycan samples were analyzed in negative ion reflectron mode. **(A)** A representative mass spectrum with annotated *N*-oligosaccharide structures is shown. **(B)** Heat map presents quantitative analysis of all glycan compositions detected by MALDI-Tof MS. **(C)** Quantitative analysis of the main glycan types: oligomannose and complex-type, and common structural features of glycans: galactosylation and fucosylation. **(D)** RT-qPCR analysis results for genes of selected glycosidases and glycosyltransferases. Results are expressed as **(B)** mean values and **(C)** mean ± SD. The statistical significance of the data was assessed using the Kruskal-Wallis test with Dunn’s correction. Significance levels between test and control groups were marked with asterisks as follows: *p ≤ 0.05, **p ≤ 0.01, ***p ≤ 0.001, and ****p ≤ 0.0001. Comparisons between test groups: HT2 and HT1, were marked with hashtags as follows # p ≤ 0.05 and #### p ≤ 0.0001. *B4GALT1*, β1,4-galactosyltransferase 1; Fuc, fucose; *FUT8*, fucosyltransferase 8; H, hexose; HT, Hashimoto’s thyroiditis; *MAN1A1*, α1,2-mannosidase IA; *MAN1A2*, α1,2-mannosidase IIA, *MAN2A1*, α1,3/1,6-mannosidase IA; *MAN2A2*, α1,3/1,6-mannosidase IIA; *MGAT1*, α1,3-mannosyl-2-β-*N*-acetylglucosaminyltransferase; *MGAT2*, α1,6-mannosyl-2-β-*N*-acetylglucosaminyltransferase; *MGAT5*, 6-β-*N*-acetylglucosaminyltransferase; N, *N*-acetylhexosamine; S, sialic acid; *ST3GAL4*, β-galactoside α2,3-sialyltransferase 4; *ST6GAL1*, sialyltransferase 1; *ST8SIA4*, α2,8-sialyltransferase 4.

**Figure 6:**
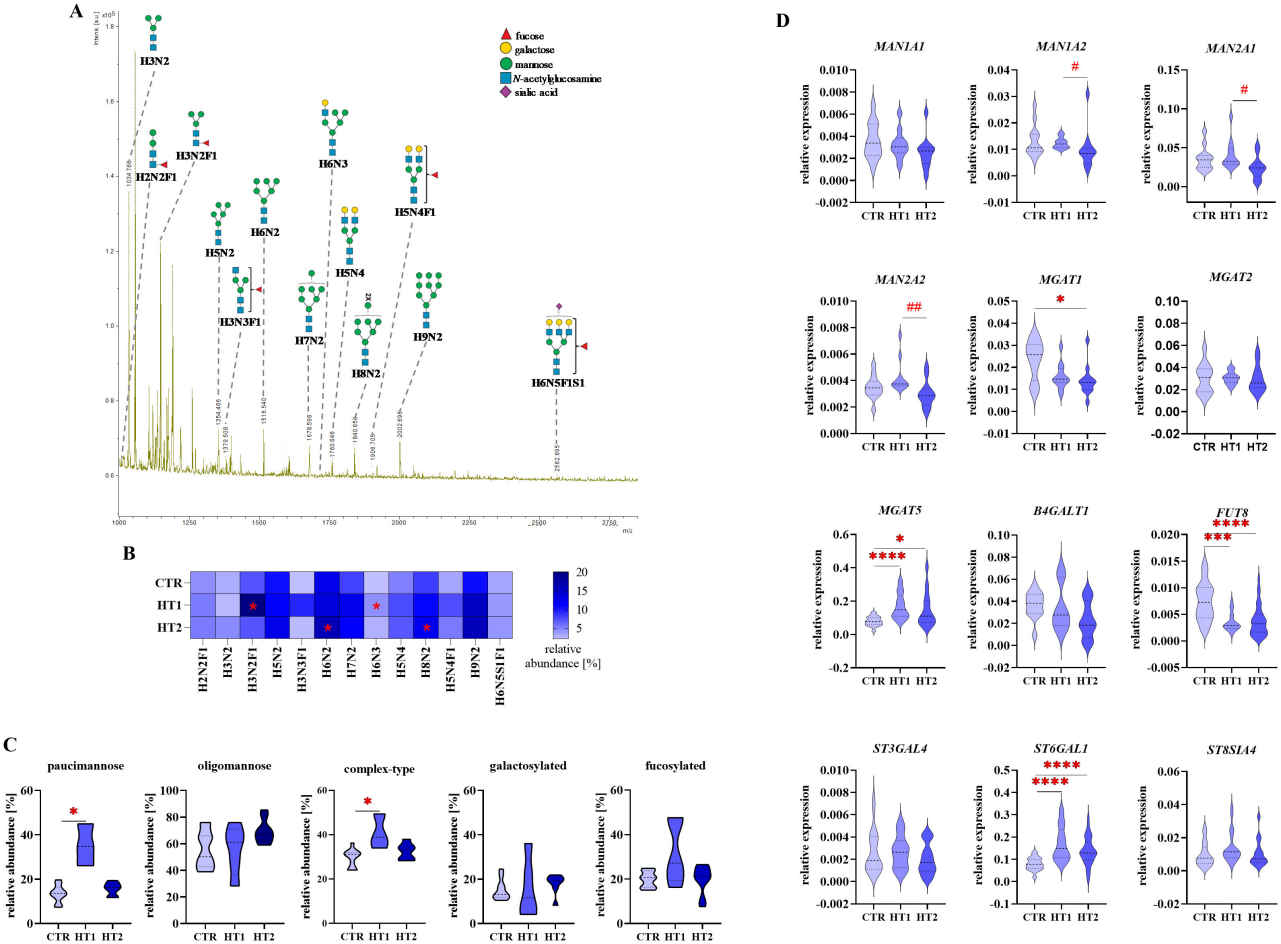
Analysis of *N*-glycan structures by MALDI-Tof MS and glycogene expression analysis by RT-qPCR in CD4^+^CD25^+^ Th cells from donors with high titers of autoantibodies without symptoms of hypothyroidism (HT1) and patients with Hashimoto’s thyroiditis during L-thyroxine therapy (HT2) and from healthy donors (CTR). *N*-glycan samples were analyzed in negative ion reflectron mode. **(A)** A representative mass spectrum with annotated *N*-oligosaccharide structures is shown. **(B)** Heat map presents quantitative analysis of all glycan compositions detected by MALDI-Tof MS. **(C)** Quantitative analysis of the main glycan types: paucimannose, oligomannose, and complex-type, and common structural features of glycans: galactosylation and fucosylation. **(D)** RT-qPCR analysis results for genes of selected glycosidases and glycosyltransferases. Results are expressed as **(B)** mean values and **(C)** mean ± SD. The statistical significance of the data was assessed using the Kruskal-Wallis test with Dunn’s correction. Significance levels between test and control groups were marked with asterisks as follows: *p ≤ 0.05, ***p ≤ 0.001, and ****p ≤ 0.0001. Comparisons between HT2 and HT1 groups were marked with hashtags as follows # p ≤ 0.05 and ## p ≤ 0.01. *B4GALT1*, β1,4-galactosyltransferase 1; Fuc, fucose; *FUT8*, fucosyltransferase 8; H, hexose; HT, Hashimoto’s thyroiditis; *MAN1A1*, α1,2mannosidase IA; *MAN1A2*, α1,2mannosidase IIA, *MAN2A1*, α1,3/1,6-mannosidase IA; *MAN2A2*, α1,3/1,6-mannosidase IIA; *MGAT1*, α1,3-mannosyl-2-β-*N*-acetylglucosaminyltransferase; *MGAT2*, α1,6-mannosyl-2-β-*N*-acetylglucosaminyltransferase; *MGAT5*, 6-β-*N*-acetylglucosaminyltransferase; N, *N*-acetylhexosamine; S, sialic acid; *ST3GAL4*, β-galactoside α2,3-sialyltransferase 4; *ST6GAL1*, sialyltransferase 1; *ST8SIA4*, α2,8-sialyltransferase 4.

**Figure 7:**
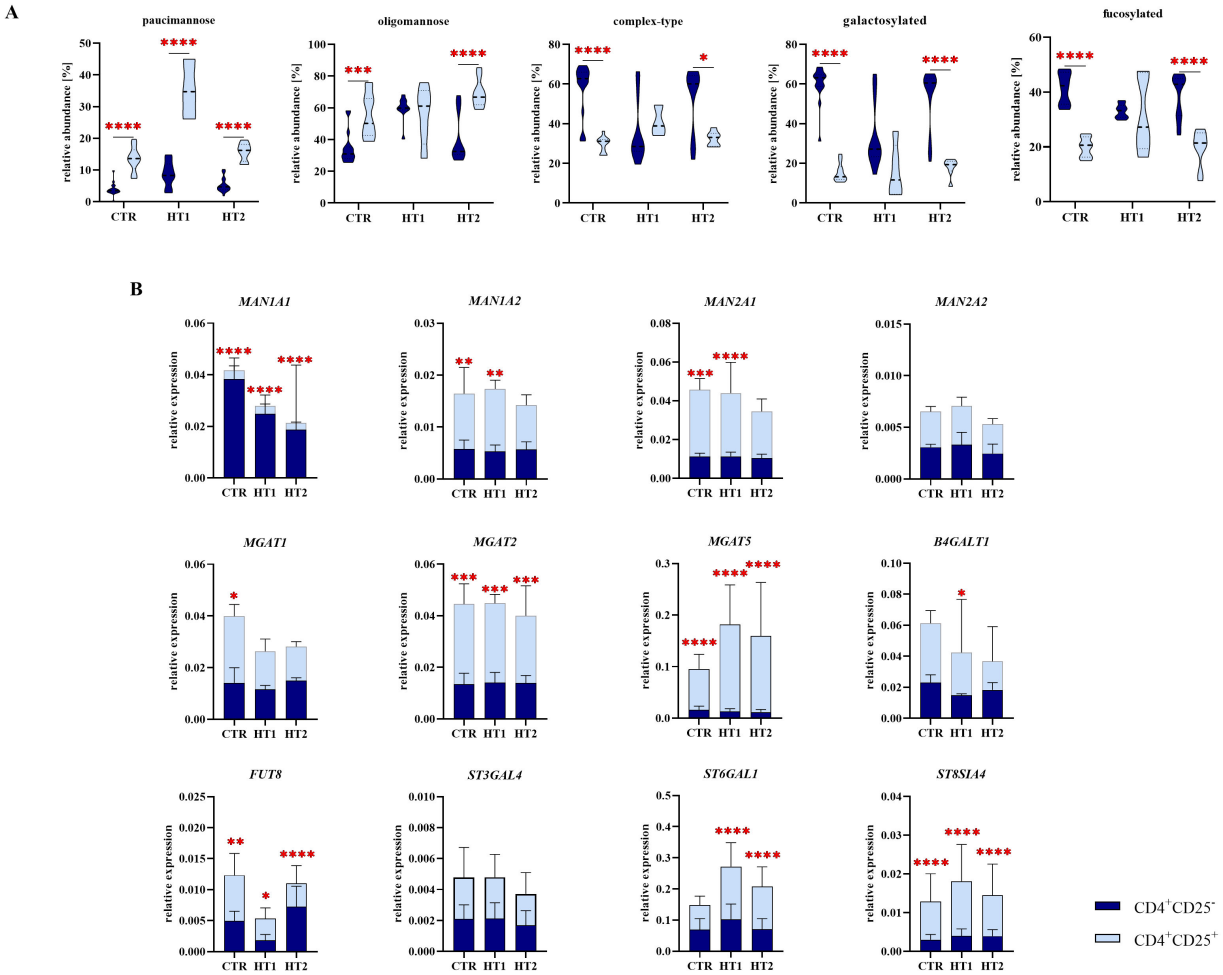
Analysis of *N*-glycan structures by MALDI-Tof MS and gene expression analysis of selected glycosidases and glycosyltransferases by RT-qPCR in CD4^+^CD25^-^ and CD4^+^CD25^+^ Th cells from patients with high titers of autoantibodies without symptoms of hypothyroidism (HT1) and subjects with Hashimoto’s thyroiditis during L-thyroxine therapy (HT2) and from healthy donors (CTR). **(A)** Comparative analysis of major *N*-glycan types: oligomannose and complex-type, and common structural features of *N*-oligosaccharides: galactosylation and fucosylation between Th cell populations. **(B)** Comparative analysis of the relative expression of selected glycosyltransferases: *MAN1A1*, *MAN1A2*, *MAN2A1*, *MAN2A2*, *MGAT1*, *MGAT2*, *MGAT5*, *B4GALT1*, *FUT8*, *ST6GAL1*, *ST3GAL4*, and *ST8SIA4* between subpopulations of Th cells. The statistical significance of the data was assessed using two-way ANOVA with Bonferroni correction. Significance levels are marked with asterisks as follows: *p ≤ 0.05, **p ≤ 0.01, ***p ≤ 0.001, and ****p ≤ 0.0001. *B4GALT1*, β1,4-galactosyltransferase 1; *FUT8*, fucosyltransferase 8; *MAN1A1*, α1,2-mannosidase IA; *MAN1A2*, α1,2-mannosidase IIA, *MAN2A1*, α1,3/1,6-mannosidase IA; *MAN2A2*, α1,3/1,6-mannosidase IIA; *MGAT1*, α1,3-mannosyl-2-β-*N*-acetylglucosaminyltransferase; *MGAT2*, α1,6-mannosyl-2-β-*N*-acetylglucosaminyltransferase; *MGAT5*, 6-β-*N*-acetylglucosaminyltransferase; *ST3GAL4*, β-galactoside α2,3-sialyltransferase 4; *ST6GAL1*, sialyltransferase 1; *ST8SIA4*, α2,8-sialyltransferase 4.

To assess changes in *N*-glycosylation, structural analysis was performed using the MALDI-Tof MS for 2-AA-labeled *N*-oligosaccharides derived from Th lymphocytes, and RT-qPCR was applied to determine the expression of glycogenes (**Figures 5**-**7**). Some of the possible isoforms of *N*-linked glycans are shown in representative mass spectra (**Figures 5A**, **6A**, and **Supplementary Tables 1**, **2**). *N*-glycans were analyzed individually (**Figures 5B**, **6B**), as well as by distinguishing groups of structures: paucimannose, oligomannose, complex-type, galactosylated, and fucosylated (**Figures 5C**, **6C**). The expression of genes encoding α-mannosidases was determined: *MAN1A1*, *MAN1A2* removing α1,2-linked mannose and *MAN2A2* detaching α1,3/α1,6-linked mannose, and glycosyltransferases: *B4GALT1* attaching galactose with a β1,4 glycosidic linkage, *MGAT1*, *2* and *5* responsible for elongating glycans with β1,2 and β1,6 antennae, *FUT8* attaching α1,6-fucose to the core GlcNAc, and sialyltransferases: *ST3GAL4*, *ST6GAL1* and *ST8SIA4* linking sialic acid with α2,3-, α2,6- and α2,8-glycosidic bonds, respectively (**Figures 5D**, **6D**, **7**).

### 3.1 *N*-glycosylation profiles of CD4^+^CD25^-^ T cells

MALDI-Tof MS analysis defined 20 different peaks in the mass spectra corresponding to *N*-oligosaccharide structures of CD4^+^CD25^-^ cells. MS data are summarized in **Figures 5A–C** and **Supplementary Table 1**, in which Symbol Nomenclature for Glycans (SNFG) was used to draw *N*-glycans.

In CD4^+^CD25^-^ T cells, we observed the up-regulation of H3N2 and H3N2F1 (*m/z* 1030.30 and 1176.34) paucimannosidic structures and H6N2 and H7N2 (*m/z* 1516.42 and 1678.46) oligomannosidic *N*-glycans, together with a decrease of galactosylated oligosaccharide H5N4 (*m/z* 1760.51) in patients from both study groups (HT1 and HT2) relative to CTR. Three other oligomannose-type *N*-glycans (H4N2, *m/z* 1192.33; H5N2, *m/z* 1354.38 and H9N2, *m/z* 2002.55) were up-regulated only in HT1 subjects compared to healthy donors. The content of fucosylated oligosaccharides (H4N4F1, *m/z* 1744.52 and H5N4F1, *m/z* 1906.56) was lower in the HT1 group vs. CTR. In the HT2 group, we observed an increase in the percentage of complex-type glycans H6N5 and H7N6 (*m/z* 2125.62 and 2490.73) vs. CTR, and a decrease in the paucimannose structure H3N2F1 (*m/z* 1176.34) and oligomannose H4N2 and H5N2 (*m/z* 1192.33 and 1354.38) vs. HT1 (**Figure 5B**). The results for the *N*-oligosaccharide subgroups showed a significant up-regulation of paucimannose- and oligomannose-type *N*-glycans, accompanied by a down-regulation of complex-type structures, including a pool of galactosylated and fucosylated glycoforms in HT1 donors compared to healthy volunteers. These changes were to some extent reversed in the HT2 group, which showed the lower content of oligomannose structures and the higher level of galactosylation vs. HT1. What is important, in all analyzed *N*-glycan subgroups in HT2 donors, we observed the tendency to restore their control-like levels (**Figure 5C**).

We did not find the changes in the expression of the selected genes encoding α-mannosidases in HT1 vs. CTR group, and HT2 vs. HT1 (**Figure 5D**), which means that the observed significant increase in the amount of oligmannose forms followed by the normalization of their content in HT2 patients (**Figure 5C**) results probably from the altered expression of other α-mannosidases or the changed activity of the studied enzymes. An attenuation of complex-type *N*-glycans, including galactosylated forms in HT1 group vs. CTR, and their reconstruction in HT2 (**Figure 5C**) is reflected in the lower *MGAT1* and *B4GALT1* expression in HT1 and the intensified *MGAT1* expression in HT2, respectively (**Figure 5D**). Not statistically significant up-regulation of complex-type *N*-glycans in HT2 vs. HT1 donors (**Figure 5C**) may result from differently regulated expression of the enzymes involved in this step of glycan processing. Formation of complex-type structures depends on a set of different glycosyltransferases, among others, the analyzed *MGAT1* and *MGAT2* responsible for the early enzymatic reactions, and *MGAT5*, whose product, *N*-acetylglucosaminyltransferase-V (GnT-V), acts at a later stage of complex-type synthesis. We identified the higher expression of *MGAT1* in HT2 vs. HT1, no changed expression of *MGAT2*, and a lower expression of *MGAT5* in HT2 vs. CTR. (**Figure 5D**). MS result for fucosylated *N*-oligosaccharides reduced in HT1 vs. CTR (**Figure 5C**) correlates with the change of *FUT8* gene expression (**Figure 5D**). Significant up-regulation of *FUT8* expression in HT2 in relation to the HT1 group was not reflected in the level of fucosylated *N*-glycans, which may be modified by other fucosyltransferase with diverse specificity, differently regulated in HT. Due to the used procedure, the recovery of sialylated structures was not high, and we detected only one sialylated *N*-glycan (H7N6S1F1, *m/z* 2562.87) which content was stable between the groups. We were able to perform the genetic analysis for the three main sialyltransferases, whose expression has been shown in Th cells previously (15, 16). Among them, *ST6GAL1* transcription was up-regulated in the HT1 group relative to CTR and normalized to the level of control in HT2 (**Figure 5B**).

### 3.2 *N*-glycosylation profiles of CD4^+^CD25^+^ T cells

In the mass spectra for *N*-glycans of CD4^+^CD25^+^ cells obtained from MALDI-Tof MS, 13 glycan peaks were detected. The data from this analysis are summarized in **Figures 6A–C** and **Supplementary Table 2**.

Quantitative analysis of individual glycan structures showed a significant increase of paucimannose H3N2F1 (*m/z* 1176.34) and hybrid-type H6N3 (*m/z* 1719.48) *N*-glycans in HT1 relative to CTR, and two oligomannose structures (H6N2, *m/z* 1516.42 and H8N2, 1840.51) in HT2 vs. CTR (**Figure 6B**). Analysis of oligosaccharide subgroups demonstrated a significant up-regulation of paucimannose and complex-type structures in HT1 donors compared to healthy subjects. In both subgroups, the content of glycans in HT2 patients lowered almost to the level of the CTR group. The differences between groups in oligomannose, galactosylated, and fucosylated pools of *N*-glycans were not statistically significant (**Figure 6C**).

RT-qPCR gene analysis revealed that the expression of three α-mannosidases (*MAN1A2*, *MAN2A1*, *MAN2A2*) was significantly lower in CD4^+^CD25^+^ cells from HT2 patients in comparison to HT1 donors (**Figure 6D**). This may be a reason for the higher content of H8N2 *N*-glycan as found by MALDI-Tof MS (**Figure 6C**). Among the four analyzed glycosyltransferases responsible for the formation of complex-type structures, we observed the down-regulation of *MGAT1* expression in HT2 vs. CTR, and upregulation of *MGAT5* in both HT1 and HT2 groups. In HT2, the expression of *MGAT5* was partially restored to the level of CTR. The strongly reduced expression of *FUT8* and the enhanced level of *ST6GAL1* in both HT1 and HT2 groups vs. CTR were also documented in the gene expression analysis (**Figure 6D**).

Based on the above results, we defined the CD4^+^CD25^–^ and CD4^+^CD25^+^-specific *N*-glycomic profiles. In homeostasis (the CTR group here), a predominance of complex-type glycans was observed in CD4^+^CD25^-^ cells (60%), whereas the oligomannose-type structures prevail in CD4^+^CD25^+^ lymphocytes (55%) (**Figure 7A**). In autoimmunity and progressive thyroid dysfunction, we observed the rearrangement of *N*-glycan structures in Th cells, in opposite directions in the analyzed CD4^+^ pools. Complex-type structures are replaced by oligomannose forms in CD4^+^CD25^-^ cells from donors with significantly elevated thyroid autoantibodies (HT1). The hyperthyroidic patients (HT2), showed a restoration of glycosylation profile in these cells to the level of healthy donors (**Figure 5C**). CD4^+^CD25^+^ cells displayed an acceleration of complex-type synthesis in the HT1 group, which was normalized in HT2 donors, representing the advanced stage of autoimmunity (**Figure 6C**). The percentage of oligomannose *N*-glycans in CD4^+^CD25^+^ cells from Hashimoto’s thyroiditis patients was about twice as high as in the CD4^+^CD25^-^ pool, while complex-type glycans were in HT2 group significantly more abundant in CD4^+^CD25^-^ cells than in CD4^+^CD25^+^ (**Figure 7A**). The expression of the majority of the analyzed genes was twice (*MAN1A2*, *MAN2A1*, *MGAT1*, *BGALT1*, *FUT8*, *ST6GAL1*) or more than twice (*MAN2A1*, *MGAT2*, *MGAT5*, *ST8SIA4*) as high in CD4^+^CD25^+^ cells compared to CD4^+^CD25^-^ cells in at least one analyzed group (**Figure 7B**).

## 4 Discussion

*N*-glycans are involved in fundamental cellular and molecular processes that stimulate and inhibit immune pathways. Changes in the glycosylation of CD4^+^ T cells regulate important pathophysiological steps such as selection, signaling, differentiation, and proliferation. Disruption of these mechanisms leads to loss of immune tolerance and overreactivity of the immune system, resulting in the development of autoimmune diseases (17).

To date, studies of glycoimmunology in Hashimoto’s thyroiditis have focused on proteins isolated from patients’ sera, mainly immunoglobulin G (IgG). One of the first studies documented that the *N*-glycan sialylation of TgAb antibodies was lower in HT patients than in patients with Graves’ disease (GD) and papillary thyroid cancer (PTC) (18). Moreover, the content of mannose and sialic acid in TgAb increased in HT compared to healthy subjects (19). Our previous study showed that hypothyroidism in HT (HT2 group) was characterized by down-regulation of F(6)A2G(4)2 and F(6)A2G(4)2S core fucosylated IgG *N*-glycans, and up-regulation of F(6)A2 *N*-glycan compared to the patients with the elevated autoantibodies titers and proper thyroid function (HT1 group) (20). Martin et al. revealed reduced α1,6-fucosylation of IgG in HT patients compared to healthy controls, which correlated with serum TPOAb titers (21). Glycosylation changes in thyroid autoimmunity were also detected in serum glycoproteins other than IgG. Removing IgGs allows the detection of *N*-glycosylation changes in proteins that are present at lower concentrations in serum. An increase in the pool of di- and triantennary complex-type glycan structures with attached sialic acid was observed in IgG-depleted sera from HT in relation to healthy donors (22). Interestingly, changes in *N*-glycosylation of thyroid follicular cells from HT patients were also identified. Thyroid biopsy scrapings were analyzed using lectin histochemistry. *Sambucus nigra* agglutinin (SNA) staining of thyroid sections showed stronger α2,6-sialylation in thyrocytes from HT subjects relative to controls. *Tritrichomonas mobilensis* lectin (TML), with wider specificity due to a recognition of all sialic acids, binds more strongly to the lymphocytes infiltrating the thyroid gland than to thyrocytes from HT patients. This proves that autoimmunity is accompanied by glycosylation changes not only in immune system glycoconjugates but also in target cells (23).

Studies on Th cell glycoimmunology have so far been performed to a limited extent in autoimmune diseases, and never in HT. The present study is a continuation of our previous analyses of HT serum proteins (22), including IgG (20, 21), and GD Th cells (24), and focuses on the *N*-linked glycosylation of CD4^+^CD25^-^ and CD4^+^CD25^+^ T cells in HT autoimmunity.

One of the best-studied types of glycosylation in the T cell is mannosylation. In the presence of cyclosporin A (CsA) and rapamycin (Rapa), immunosuppressive drugs used in transplantation, we demonstrated changes in the percentage of oligomannose-type *N*-glycans in PBMCs, which include Th cells. Using a two-way mixed leukocyte response (MLR) model, CsA was shown to down-regulate PBMC mannosylation, while the synergistic effect of Rapa and CsA up-regulated the pool of oligomannose structures (25). Oligomannose-type *N*-oligosaccharides are essential for T cell biology and function. *N*-glycosylation of thymocytes was inhibited at the level of *N*-oligosaccharides rich in mannose residues in the *Rag1^Cre^Mgat1^fl/fl^* mouse model. Thus, mannosylation of thymocytes was observed to limit their maturation, activation, passage of positive/negative selection, and differentiation into a population of Treg cells. As a consequence, mice showed increased susceptibility to nephritis and colitis (26). In the present study, we found a higher oligomannose glycan pool (**Figure 5C**), and up-regulated individual oligomannosidic structures (H4N2, H5N2, H6N2, H7N2, and H9N2) (**Figure 5B**) in CD4^+^CD25^-^ cells from HT1 donors compared to healthy controls. Interestingly, total mannosylation (**Figure 5C**) and the content of H4N2 and H5N2 structures (**Figure 5B**) in the CD4^+^CD25^-^ cells from HT2 patients, resembled the levels of healthy controls. CD4^+^CD25^+^ cells in HT2 had significantly more oligomannose *N*-glycans than healthy subjects (**Figure 6C**), and twice as many as HT2 CD4^+^CD25^-^ cells (**Figure 7A**). Thus, we speculate that oligomannose-type structures may be essential for Th cell activation during thyroid autoimmunity. The abundant pool of oligomannose glycoforms in CD4^+^CD25^+^ cells correlated with the lower expression of the glycogenes encoding α-mannosidases (**Figure 6D**). Thus, these results suggest a regulatory importance of Th cells’ mannosylation in Hashimoto’s thyroiditis, which has not been described so far. A shifted ratio of complex-to-oligomannose *N*-glycans was also found in Graves’ disease, where complex-type structures were partially replaced by oligomannose forms in CD4^+^CD25^-^ in patients compared to healthy subjects (24). An enrichment of oligomannose glycans at the expense of complex-type structures as a result of the impaired *MAN2A1* expression was previously demonstrated in lupus nephritis by Alves et al. (27). Replacing branched *N*-glycans with oligomannose glycoforms on T cells was recognized as a part of the loss of their regulatory mechanism dependent on galectins, which led to activation of a pro-inflammatory phenotype (28).

The restoration of oligomannose-type and galactosylated complex-type *N*-glycans in the HT2 group to levels observed in the CTR group (**Figure 5C**) is intriguing, and the underlying mechanism warrants further investigation. Thyroid hormones were shown to modulate the metabolism and the activity of immune cells, including CD4^+^ T cells (29, 30). In our study, the values of TSH were in a reference range in each group (**Figure 1**), meaning that the levels of thyroid hormones were also not altered. HT2 patients were supplemented with the physiological doses of L-thyroxine (T4) to the endogenous level of T4 in CTR and HT1 donors. Thus, our finding on normalization of the major *N*-glycan types in Hashimoto’s thyroiditis patients does not seem to depend on thyroid hormones, but rather on the changes in inflammation after normalization of thyroid hormone levels.

The present analysis demonstrated a significantly decreased *MGAT5* expression in CD4^+^CD25^-^ cells (**Figure 5D**), and a higher level of *MGAT5* transcript in CD4^+^CD25^+^ cells from HT patients compared to healthy subjects (**Figure 6D**). Importantly, *MGAT5* expression was 2-fold higher in CD25^+^ activated cells than in non-activated cells (**Figure 7B**). Evaluation of *MGAT5* expression and its β1,6-GlcNAc branched glycan products has been well described in human multiple sclerosis (MS) and experimental autoimmune encephalitis (EAE) in mice. Both in MS and EAE, *MGAT5* expression and the synthesis of *N*-glycans with β1,6-GlcNAc antenna negatively correlated with disease progression (31). Genetically modified *Mgat5^-/-^* mice were observed to be more susceptible to EAE and its more severe course, resulting from spontaneous neurodegeneration. β1,6-GlcNAc-branched *N*-oligosaccharides present on Th cell surface receptors blocked Th1 cell differentiation and increased polarization toward Th2 cells involved in humoral-type immune responses (32–34). In addition, in *Mgat5^-/-^* knockout mice, due to inhibition of β1,6-GlcNAc branching, clustering of T cell receptors (TCRs) within the plasma membrane was enhanced, resulting in a reduction in Th lymphocyte excitability and excessive activation characteristic of the autoimmune process (31). The lowered *MGAT5* expression in CD4^+^CD25^-^ cells in the HT2 group vs. CTR (**Figure 5D**) was not reflected in the level of complex-type *N*-glycans analyzed individually and as a group (**Figures 5B, C**). More detailed analysis will be necessary to detect β1,6-GlcNAc-branched structures among the identified complex-type *N*-glycans, not possible at this stage of research due to the limited amount of protein extracted from isolated Th cells. Our data indicate an up-regulation of *MGAT5* expression in activated CD25^+^ Th cells, especially higher in HT1 donors, and to a lesser extent also in HT2 patients. Among Th CD4^+^CD25^+^, regulatory T cells (Tregs), a subset of Th cells with a low level of CD127 and the expression of Forkhead box P3 transcription factor (FOXP3) (CD127^low^ FOXP3^+^) play a unique suppressive role in T cell activity. Treg expression was found not to be changed in autoimmune thyroid diseases (AITDs), but their function was attenuated in thyroid autoimmunity (35). Branched *N*-glycans in Tregs seem to be crucial for their suppressive role towards the rest of T cells (36). The up-regulated *MGAT5* expression in both HT1 and HT2 groups vs. CTR (**Figure 6D**) and the higher content of complex-type *N*-glycans in HT1 (**Figure 6C**) suggest the importance of branched glycans in the CD25^+^ pool of CD4^+^ cells during the early stage of autoimmunization. In chronic inflammation, specific for HT2 patients, the impaired activity of Tregs, shown previously by Glick et al. (35), may result, among others, from their altered *N*-glycosylation.

Fucosylation changes in T cells during hyperactivation and ongoing autoimmunity have been studied to a limited extent. Liang et al, using a mouse model of EAE with *Fut8^-/-^* knockout, showed that reduction of core fucosylation significantly attenuated disease symptoms and CD4^+^ T cell proliferation. A lack of core fucosylation increased T cell activation through TCR interactions with MHC II on the surface of B cells (37). Core α1,6-fucose is required for the induction of TCR-anti-CD3/CD28 signalling leading to inflammation in patients with inflammatory bowel disease (IBD) (38). CD4^+^ T cells from patients with systemic lupus erythematosus (SLE) showed an elevated core α1,6-fucosylation compared to healthy individuals (37). Our results revealed the significantly reduced level of total fucosylation (**Figure 5C**) and the amount of fucosylated H4N4F1 and H5N4F1 structures (**Figure 5B**) in CD4^+^CD25^-^ from HT1 donors vs. CTR, which correlated with lower *FUT8* expression (**Figure 5D**). In HT2 patients, *FUT8* expression was upregulated compared to the HT1 group and slightly above the level of CTR (**Figure 5D**), which was reflected in the higher amount of fucosylated glycans (**Figure 5C**). CD4^+^CD25^+^ cells had significantly reduced expression of the *FUT8* transcript in both HT groups compared to the control group (**Figure 6D**). To understand the impact of the alterations of fucosylation in Th cells, which accompany inflammatory and autoimmune processes, further studies are needed.

Immunophenotyping for the entire pool of Th CD69^+^ lymphocytes showed significant differences in the percentage of these cells in HT1 and HT2 patients compared to healthy subjects (**Figure 2B**), which was noted in previously published studies (39). This underscores the key role of the Th cell group in thyroid autoimmunity.

In conclusion, our study demonstrates for the first time that *N*-glycosylation of CD4^+^ cells is subject to a fundamental change in Hashimoto’s thyroiditis. Th cells alter their *N*-glycan synthesis early when thyroid autoantibodies are produced, and the thyroid gland does not show symptoms of hypothyroidism. Development of HT, defined by both the presence of autoantibodies and hypothyroidism, is characterized by disease-specific changes of T cell *N*-glycome, different from the *N*-glycosylation profile at the previous stage, with the elevated autoantibodies. An important finding in our research is a variation of T cell glycosylation dependent on their activation, defined by the expression of CD25 protein. *N*-glycosylation of a pool of CD25^+^ Th cells is differently changed in HT than the rest of the CD4^+^ cells without this marker. Due to the heterogeneity of the Th lymphocyte subsets, assigning the observed changes to a given Th cell population is necessary as a continuation of the present research. Knowing the importance of *N*-glycans in protein structure and functions, it seems very likely that the detected *N*-glycan rearrangement in Th cells contributes to the breakdown of immune tolerance and hyperactivation in thyroid autoimmunity. Further studies are needed to determine how the overexposed glycomic changes affect T cell proliferation, activation, and apoptosis, or the function of the thyrocytes themselves after contact with autoreactive CD4^+^ lymphocytes or the cytokines they secrete.

## Data Availability

The original contributions presented in the study are included in the article/**Supplementary Material**. Further inquiries can be directed to the corresponding author.

## Supplementary material

Supplementary Table 1 Annotation of MALDI-Tof MS *N*-glycans peaks for CD4^+^CD25^-^ T cells obtained from donors with high titers of TPOAb and/or TgAb autoantibodies and TSH level in the normal range without symptoms of hypothyroidism (HT1) and patients with Hashimoto’s thyroiditis with high levels of antithyroid antibodies, advanced thyroid destruction and stabilized TSH level following L-thyroxine therapy (HT2) (study groups), and from healthy subjects (CTR, control group). *N*-oligosaccharides were identified based on *m/z* values measured in negative ion reflectron mode and common knowledge of glycobiology. Observed *m/z* values were calculated as averages for all analyzed samples. *N*-glycan structures in Symbol Nomenclature for Glycans (SNFG) notation were prepared in GlycoWorkbench software (version 2.1. European Carbohydrates DataBase project; http://www.eurocarbdb.org/). In some cases, more structural isoforms are possible. CT, complex-type *N*-glycans; F, fucose; Fuc, fucosylated *N*-glycans; Gal, galactosylated *N*-glycans; H, hexose; HT, hybrid-type *N*-glycans; N, *N*-acetylhexosamine; OM, oligomannose *N*-glycans; PM, paucimannose *N*-glycans; S, sialic acid.

Supplementary Table 2 Annotation of MALDI-Tof MS *N*-glycans peaks for CD4^+^CD25^+^ T cells obtained from donors with high titers of TPOAb and/or TgAb autoantibodies and TSH level in the normal range without symptoms of hypothyroidism (HT1) and patients with Hashimoto’s thyroiditis with high levels of antithyroid antibodies, advanced thyroid destruction and stabilized TSH levels following L-thyroxine therapy (HT2) (study groups) and from healthy subjects (CTR, control group). *N*-oligosaccharides were identified based on *m/z* values measured in negative ion reflectron mode and common knowledge of glycobiology. Observed *m/z* values were calculated as averages for all analyzed samples. *N*-glycan structures in Symbol Nomenclature for Glycans (SNFG) notation were prepared in GlycoWorkbench software (version 2.1. European Carbohydrates DataBase project; http://www.eurocarbdb.org/). In some cases, more structural isoforms are possible. CT, complex-type *N*-glycans; F, fucose; Fuc, fucosylated *N*-glycans; Gal, galactosylated *N*-glycans; H, hexose; HT, hybrid-type *N*-glycans; N, *N*-acetylhexosamine; OM, oligomannose *N*-glycans; PM, paucimannose *N*-glycans; S, sialic acid.
