# Supplementary material for: The diverse N-glycosylation profiles of CD4+CD25- and CD4+CD25+ T cells in Hashimoto’s thyroiditis
Source: Front Immunol. 2025 Sep 15;16:1633344. doi: 10.3389/fimmu.2025.1633344 (PMC12477247; doi:10.3389/fimmu.2025.1633344)
Supplement: Supplementary Table 1 — Annotation of MALDI-Tof MS N-glycans peaks for CD4+CD25- T cells obtained from donors with high titers of TPOAb and/or TgAb autoantibodies and TSH level in the normal range without symptoms of hypothyroidism (HT1) and patients with Hashimoto’s thyroiditis with high levels of antithyroid antibodies, advanced thyroid destruction and stabilized TSH level following L-thyroxine therapy (HT2) (study groups), and from healthy subjects (CTR, control group). N-oligosaccharides were identified based on m/z values measured in negative ion reflectron mode and common knowledge of glycobiology. Observed m/z values were calculated as averages for all analyzed samples. N-glycan structures in Symbol Nomenclature for Glycans (SNFG) notation were prepared in GlycoWorkbench software (version 2.1. European Carbohydrates DataBase project; http://www.eurocarbdb.org/). In some cases, more structural isoforms are possible. CT, complex-type N-glycans; F, fucose; Fuc, fucosylated N-glycans; Gal, galactosylated N-glycans; H, hexose; HT, hybrid-type N-glycans; N, N-acetylhexosamine; OM, oligomannose N-glycans; PM, paucimannose N-glycans; S, sialic acid. [file Table1.docx]

Supplementary Material

# Supplementary Table 1. Annotation of MALDI-Tof MS *N*-glycans peaks for CD4^+^CD25^-^ T cells obtained from donors with high titers of TPOAb and/or TgAb autoantibodies and TSH levels in the normal range without symptoms of hypothyroidism (HT1) and patients with Hashimoto's thyroiditis with high levels of antithyroid antibodies, advanced thyroid destruction and stabilized TSH levels following L-thyroxine therapy (HT2) (study groups), and from healthy subjects (CTR, control group). *N*-oligosaccharides were identified based on *m/z* values measured in negative ion reflectron mode and common knowledge of glycobiology. Observed *m/z* values were calculated as averages for all analyzed samples. *N*-glycan structures in Symbol Nomenclature for Glycans (SNFG) notation were prepared in GlycoWorkbench software (version 2.1. European Carbohydrates DataBase project; http://www.eurocarbdb.org/). In some cases, more structural isoforms are possible. CT, complex-type; F, fucose; Fuc, fucosylated; Gal, galactosylated; H, hexose; HT, hybrid-type; N, *N*-acetylhexosamine; OM, oligomannose; PM, paucimannose; S, sialic acid.

| Peak ID | Component name | *N*-glycan structure type | Theoretical *m/z* | Observed *m/z* (average) | | *N*-glycan structure (SNFG notation) |
| --- | --- | --- | --- | --- | --- | --- |
| 1 | H3N2 | PM | 1030.37 | 1030.30 | 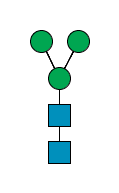 | |
| 2 | H3N2F1 | PM, Fuc | 1176.43 | 1176.34 | 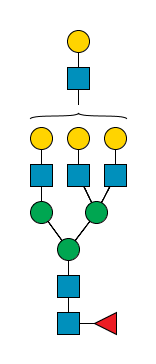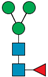 | |
| 3 | H4N2 | OM | 1192.42 | 1192.33 | 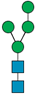 | |
| 4 | H5N2 | OM | 1354.47 | 1354.38 | 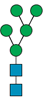 | |
| 5 | H3N3F1 | CT, Fuc | 1379.41 | 1379.41 | 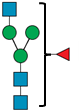 | |
| 6 | H6N2 | OM | 1516.53 | 1516.42 | 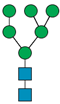 | |
| 7 | H4N3F1 | CT, Fuc | 1541.56 | 1541.45 | 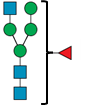 | |
| 8 | H7N2 | OM | 1678.58 | 1678.46 | 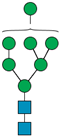 | |
| 9 | H6N3 | HT, Gal | 1719.61 | 1719.48 | 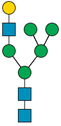 | |
| 10 | H4N4F1 | CT, Gal, Fuc | 1744.64 | 1744.52 | 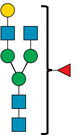 | |
| 11 | H5N4 | CT, Gal | 1760.63 | 1760.51 | 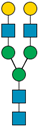 | |
| 12 | H8N2 | OM | 1840.63 | 1840.51 | 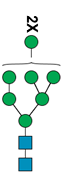 | |
| 13 | H5N4F1 | CT, Gal, Fuc | 1906.69 | 1906.56 | 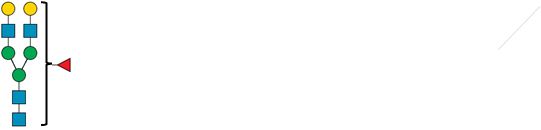 | |
| 14 | H9N2 | OM | 2002.69 | 2002.55 | 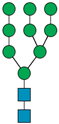 | |
| 15 | H6N5 | CT, Gal | 2125.76 | 2125.62 | 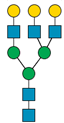 | |
| 16 | H6N5F1 | CT, Gal, Fuc | 2271.82 | 2271.67 | 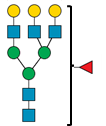 | |
| 17 | H6N5F2 | CT, Gal, Fuc | 2417.88 | 2418.72 | 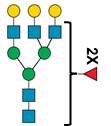 | |
| 18 | H6N6F1 | CT, Gal, Fuc | 2474.90 | 2474.75 | 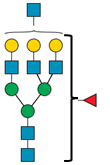 | |
| 19 | H7N6 | CT, Gal | 2490.90 | 2490.73 | 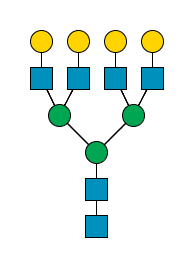 | |
| 20 | H6N5F1S1 | CT, Gal, Fuc | 2562.92 | 2562.87 | 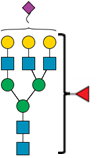 | |
